# Continuous Monitoring and Risk Assessment of Multiple Pesticide Residues in Pineapple

**DOI:** 10.3390/foods14233983

**Published:** 2025-11-21

**Authors:** Cheng Luo, Ru Zhou, Shaodong Zeng, Qi Li, Ling Lin, Yalin Zhang, Jianzhi Ye, Yarong Zhao

**Affiliations:** 1 Agricultural Products Processing Research Institute, Chinese Academy of Tropical Agricultural Sciences, Zhanjiang 524000, China; 2 Laboratory of Quality & Safety Risk Assessment on Agro-Products Processing (Zhanjiang), Ministry of Agriculture and Rural Affairs, Zhanjiang 524000, China; 3 Institute of Quality Standard and Monitoring Technology for Agro-Products of Guangdong Academy of Agricultural Sciences, Guangzhou 510640, China

**Keywords:** pineapple, risk assessment, monitoring, pesticides

## Abstract

Pesticide residues are one of the key factors affecting food safety and human health. This study systematically monitored the pesticide residue levels in pineapples from major production regions in China and assessed the dietary exposure risks. A total of 387 samples were collected during the pineapple harvesting seasons from 2023 to 2025. The residues of 88 pesticides were detected using GC, GC-MS, and LC-MS/MS. The results showed that 20 pesticides were detected, with detection rates of cypermethrin, carbendazim, and gibberellic acid exceeding 10%. Based on the Chinese National Standard GB 2763, the compliance rate of the samples was 98.71%. In the worst-case scenario, the ADI % for all population subgroups was less than 1%, the %ARfD was less than 60%, and the Hazard Index was less than 1.5%, far below the 100% risk threshold, indicating that the pesticide residue risk in pineapples is low and at an acceptable level.

## 1. Introduction

Chemical agents constitute fundamental elements within current agricultural systems, proving indispensable for securing harvest-viable quantities and characteristics of economic plant species [1]. However, the improper use of pesticides may lead to their residues in agricultural products and the environment, thus posing potential food safety risks. Globally, pesticide residue risk assessment has become a key issue in the field of food safety, aiming to safeguard consumer health and maintain fair trade [1,2,3].

In the southern regions of China, Ananas comosus is formally categorized into the “Four Major Fruits of Southern China,” together with bananas (*Musa acuminata*), lychees (*Litchi chinensis*), and longans (*Dimocarpus longan*). To date, its cultivation scope has expanded to more than 80 countries and regions worldwide, forming a large-scale industrial layout with geographical integration characteristics. According to the 2024 statistical bulletin issued by the Tropical Crop Center of the Ministry of Agriculture and Rural Affairs, the annual output of Ananas comosus in China has surpassed 2 million tons [4]. From the perspective of regional production distribution, Guangdong Province functions as the dominant Ananas comosus producing area in China, with Hainan Province ranking as the secondary production hub. The combined output of these two provinces constitutes approximately 89.7% of the national total yield, forming a distinct “dual-core” geographical distribution pattern within China’s Ananas comosus industrial sector.

Nonetheless, the deficiency of standardized management protocols in current Ananas comosus cultivation practices has emerged as a critical bottleneck constraining the high-quality development of the industry. Specifically, the pest and disease management paradigm characterized by “prioritizing post-infestation control over pre-infestation prevention” and the non-standard agricultural operation of “blindly elevating pesticide application dosages” have collectively led to a significant increase in the risk of excessive pesticide residues in Ananas comosus fruits. For pineapple crops, previous studies have indicated that pesticides used during the production process may pose diffusion risks. Individual exposure studies have shown that pesticide exposure levels in residential areas surrounding pineapple plantations (especially among children and adolescents) are significantly correlated with proximity to local intensive farming practices [5]. The evidence reveals that agrochemicals utilized within pineapple farming operations can spread beyond restricted agricultural areas, posing possible translocation threats to surrounding habitats and individual exposure.

As a popular tropical fruit, the pesticide residue levels in pineapples are directly related to consumer dietary safety. The US Environmental Working Group (EWG) has classified pineapple as a ‘cleaner fruit and vegetable’, though it still contains low levels of thiamethoxam residue—a substance confirmed to disrupt children’s nervous system development. Organochlorine pesticide residues were identified within fruits (including pineapples) across the Ghana region, and corresponding health risk assessments have been conducted [6]. China Xiamen inspection found 0.14 mg/kg of dimethoate residue in Taiwanese pineapples, far exceeding China’s limit standard of 0.02 mg/kg. According to the study by Yiran Liang et al. [7], when spinetoram was applied at the recommended dose of 80 mg a.i./kg and during the safety interval, the maximum total residue in pineapple flesh was 0.064 mg/kg. The calculated risk quotient (RQ) was only 17.5% (far below the safety threshold of 100%), indicating a low health risk under normal consumption.

Pineapples may also generate significant residues during the harvesting and post-harvest handling processes, which, if not properly managed, could pose indirect risks to the environment and health [8]. Therefore, conducting pesticide residue risk assessments is an essential foundation for ensuring the scientific and effective regulation of food safety [2]. The internationally accepted practice is to establish a forward-looking risk assessment process and use it as a core component of pesticide registration and pre-market evaluation [1]. This process primarily includes simulating actual pesticide application patterns through field residue trials to obtain expected residue levels, estimating the dietary exposure to pesticide residues by combining population dietary consumption data, and comparing and analyzing this data against safety thresholds (such as Acceptable Daily Intake (ADI) and Acute Reference Dose (ARfD)) [1,9,10]. The EFSA PRIMo (Pesticide Residue Intake Model), widely used by the European Union, is a computational tool that integrates residue and consumption data for dietary risk assessment and provides scientific evidence for establishing Maximum Residue Limits (MRLs) [11]. In specific assessments, it is also necessary to clarify the definition of residues (including pesticides and their metabolites) to meet the regulatory and risk assessment requirements [9].

Systematically analyzing pesticide residue levels in pineapples and their potential impact on health not only helps improve the transparency of food safety information but also provides scientific evidence for regulatory authorities to establish precise risk control measures. These measures may include setting reasonable MRLs for specific pesticides in pineapples, optimizing pesticide application guidelines, and enhancing monitoring efforts [2]. The ultimate goal is to safeguard consumer health and promote environmentally responsible growth within the pineapple industry.

The present research seeks to systematically quantify actual pesticide contamination levels within Chinese pineapples while evaluating both long-term and short-term consumption hazards across various demographic groups, aiming to deliver scientific evidence and policy guidance for quality safety regulation of pineapple products.

## 2. Materials and Methods

### 2.1. Standards, Reagents, and Chemicals

The pesticide standards for this study were obtained from the Environmental Quality Supervision and Testing Center, Ministry of Agriculture and Rural Affairs (Tianjin, China), with purity ranging from 99% to 99.9%. Based on the chemical structure of the pesticides and the detection equipment used, the standards were classified into four groups and prepared as stock solutions. The specific pesticide composition of each group’s stock solution is detailed in Appendix A.

Group 1: Includes 20 organophosphorus pesticides such as omethoate, chlorpyrifos, and acephate, prepared as stock solutions at a concentration of 10 μg/mL in acetone.

Group 2: Includes 18 organochlorine pesticides such as chlorothalonil and cypermethrin, prepared as stock solutions at a concentration of 10 μg/mL in n-hexane.

Group 3: Includes 40 pesticides such as cyromazine and carbofuran, prepared as stock solutions at a concentration of 20 μg/mL in methanol.

Group 4: Includes 10 pesticides such as fipronil and fenthion, prepared as stock solutions at a concentration of 10 μg/mL in acetone.

Acetonitrile, acetone, methanol, n-hexane, formic acid, ammonium acetate (High Performance Liquid Chromatography grade), sodium chloride (analytical grade), and dSPE (Dispersive Solid Phase Extraction) cleanup cartridges were all purchased from Anpel Laboratory Technologies (Shanghai) Inc. (Shanghai, China). The water used in the experiments was prepared using a Milli-Q water purification system (Millipore, Molsheim, France).

### 2.2. Samples

From the 2023 to 2025 pineapple harvesting seasons, a total of 387 batches of pineapple samples were procured across three successive growing seasons within principal pineapple production regions in Guangdong, Guangxi, and Hainan, China, in accordance with the Chinese agricultural standard NY/T 789 Guideline on Sampling for Pesticide Residue Analysis [12]. Of these, 108 batches were from 2023, 200 batches were from 2024, and 79 batches were from 2025. Each sample consisted of at least three whole pineapples. Sampling locations included plantations, agricultural markets, and fruit stores. All samples were randomly selected to minimize subjective bias. Only one batch of samples was collected from each production site or store during each annual sampling period. The pineapples from each batch were chopped (without peeling), blended into a homogeneous paste, and stored at −18 °C in sealed containers.

### 2.3. Sample Extraction

Based on the Chinese Agricultural Industry Standard NY/T 761-2008—Pesticide multiresidue screen methods for determination of organophosphorus pesticides, organochlorine pesticides, pyrethroid pesticides, and carbamate pesticides in vegetables and fruits [13], and optimized for this study, the specific procedure is as follows.

A sample of 10.0 g of homogenized pineapple is weighed, and 5.0 g of sodium chloride, along with 20.0 mL of acetonitrile, is added. The mixture is vortexed at 2500 rpm for 5 min, with subsequent spinning at 10,000 rpm for 5 min. The supernatant is divided into four groups, each containing 4 mL. After nitrogen evaporation to near dryness, each group is treated as follows:

For pesticides in Group 1 and Group 4: After nitrogen evaporation to near dryness, add 2.0 mL of acetone. Vortex the solution at 3000 rpm for 1 min, dissolve, filter, and collect the filtrate. The filtrate is stored at 4 °C.

For pesticides in Group 3: After nitrogen evaporation to near dryness, add 2.0 mL of a 1:1 acetonitrile-water solution. Vortex the solution at 3000 rpm for 1 min, then filter, collect the filtrate, and store it at 4 °C.

For pesticides in Group 2: After nitrogen evaporation to near dryness, add 2.0 mL of n-hexane. Vortex the solution at 3000 rpm for 1 min, then add a dispersive Solid Phase Extraction (dSPE, Florisil phase, 1 g, Supelco manufactures, Pennsylvania, PA, USA) cleanup cartridge. Vortex again at 3000 rpm for 1 min, after which perform centrifugal processing at 2500 rpm for 1 min. After filtration, collect the filtrate and store it at 4 °C.

**Figure 1:**
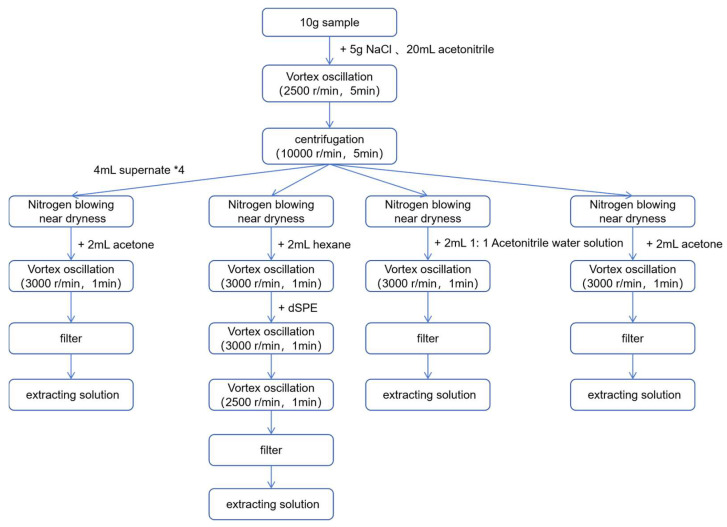
Flowchart of sample extraction process.

The complete process for sample extraction, purification, and preparation is shown in Figure 1.

### 2.4. Analytical Instruments and Conditions

#### 2.4.1. Gas Chromatography (GC) Analysis

The GC analysis method refers to China’s agricultural industry standard NY/T 761 [13].

(1) Group 1 Pesticide Analysis

The analysis was performed using a Shimadzu GC 2010 Plus system (Shimadzu Corporation, Kyoto, Japan), equipped with a Flame Photometric Detector (FPD) (Shimadzu Corporation, Kyoto, Japan) and a ZB-5MS chromatographic column (Length: 30 m, inner diameter: 0.25 mm; film thickness: 0.25 μm, phenomenex, Los Angeles, CA, USA). A programmed temperature rising split injection method was employed. The injection volume was 1 μL, with a flow rate of 1.42 mL/min and a split ratio of 4. The initial injection temperature was 140 °C, held for 2 min, then heated at 8 °C/min to 230 °C, subsequently rising at 30 °C/min to 270 °C, which was maintained for 5 min.

(2) Group 2 Pesticide Analysis

The analysis was performed using a Shimadzu GC 2010 Pro system (Shimadzu Corporation, Kyoto, Japan), equipped with an Electron Capture Detector (ECD) (Shimadzu Corporation, Kyoto, Japan) and a CD-1 chromatographic column (Length: 30 m, inner diameter: 0.25 mm; film thickness: 0.25 μm, Anpel, Shanghai, China). A programmed temperature rising non-split injection method was used. The injection volume was 1 μL, with a flow rate of 1.40 mL/min. The initial injection temperature was 150 °C, held for 2 min, followed by an increase at a rate of 15 °C/min to 270 °C, and then held for 9 min.

#### 2.4.2. Gas Chromatography–Mass Spectrometry (GC-MS) Analysis

The GC-MS analysis method refers to China’s national standard GB/T 23200.8 [14].

Group 4 Pesticide Analysis: Measurements were performed with a Shimadzu GCMS-QP2010Ultra gas chromatography–mass spectrometry system (Shimadzu Corporation, Kyoto, Japan), equipped with a DB-5MS chromatographic column (Length: 30 m, inner diameter: 0.25 mm; film thickness: 0.25 μm, Agilent, Santa Clara, CA, USA) employing electron impact ionization. A programmed temperature rising non-split injection approach was implemented. The sample injection employed a 1 μL volume at a flow velocity of 1.00 mL/min. Starting injection temperature commenced at 80 °C, was maintained for 1 min, then ramped at 40 °C/min to 200 °C, with subsequent elevation at 10 °C/min to 280 °C, where it remained for 9 min. The ion source operated at 230 °C while the interface was maintained at 280 °C. Detection was performed using the SIM mode.

#### 2.4.3. Liquid Chromatography–Mass/Mass Spectrometry (LC-MS/MS) Analysis

The LC-MS/MS analysis method refers to China’s national standard GB 23200.121 [15].

Group 3 Pesticide Analysis: Analysis proceeded using a Waters XEVO-TQD system (Waters, Milford, CT, USA) configured for electrospray ionization (ESI). Chromatographic separation was employed using a BEH C18 chromatographic column (Length: 50 mm, inner diameter: 2.1 mm, particle size: 1.7 μm, Waters, Milford, USA), operating under 40 °C thermal conditions. Two components formed the mobile phase: Phase C, which was methanol, and Phase D, which was an aqueous solution containing 0.1% formic acid and 0.05 mmol/L ammonium acetate, with a flow rate of 0.3 mL/min. The gradient elution procedure consisted of the following steps: initially 5% Phase C, which was adjusted to 95% Phase C at 2 min, further adjusted to 50% Phase C at 3.2 min, and restored to 5% Phase C at 4.5 min, maintained for 1.5 min. Sample injection employed a 3 μL volume alongside spray voltage at 0.6 kV and ion source temperature at 120 °C.

### 2.5. Risk Assessment Method

Adapted from the Veterinary Residues Committee (VRC) Veterinary Drug Residue Risk Ranking Matrix in the UK and referencing the work of Nie Jiyun et al. [16], the pharmacological indicators were replaced with toxicity indicators. The modified method was used to assess the risk levels of pesticide residues in agricultural products, with the following calculation formula:

(1) PS=(A+B)×(C+D+E+F)

in which PS denotes the pesticide risk priority value, represented by the average pesticide residue risk rating across all samples; A represents the pesticide toxicity, sourced from the China Pesticide Information Network [17]; B represents the pesticide toxicity effect (represented by ADI), sourced from the National Food Safety Standard—Maximum Residue Limits for Pesticides in Food, GB 2763 [18]; C represents the dietary proportion; D represents the frequency of pesticide usage during cultivation; E represents the high exposure population; and F represents the pesticide residue level. The scoring criteria for each index are detailed in Table 1.

To reflect pesticide residue risk in sample materials, a risk index (RI) is used to represent the risk level of each sample, with the following calculation formula:

(2) $$\mathrm{RI}=\sum \mathrm{PS}-{\mathrm{TS}}_{0}$$

where TS_0_ represents the residue risk score for samples with no detected pesticide residues.

### 2.6. Risk Assessment

The acute and chronic dietary exposure risks for pineapples are represented by dividing the pesticide residue intake by the corresponding ARfD and ADI. The relevant equations for short-term dietary intake risk assessment are presented below [19]:

(3a) $$\%ARfD=\frac{(U_{e}\times C)\times v+(LP-U_{e})\times C}{b_{w}\times ARfD}\times 100\%$$

(3b) $$\%ARfD=\frac{LP\times C\times v}{b_{w}\times ARfD}\times 100\%$$

In the equations, %ARfD denotes the exposure risk from pesticide residues within short-term consumption; LP denotes the peak large-scale portion allocation (97.5th percentile across the consumer population), quantified as kg of daily food intake; Ue denotes the edible component of unit mass, expressed in kilograms, sourced from the country that utilized the large portion, LP; v signifies the variability coefficient characterizing the ratio between residue concentrations at the 97.5th percentile and average residue levels in individual units, where v = 3; C is the maximum pesticide residue level in pineapple; and bw is the average body weight of the consuming population. The dietary intake information originates from the official WHO website [20]. The ARfD parameter is derived through the Joint Meeting on Pesticide Residues (JMPR) database [21]. The immediate dietary exposure threat for children is determined via Formula (3a), and for adults, it is calculated using Formula (3b) [22]. Should %ARfD stay beneath 100, the expected rapid threat is viewed as satisfactory. On the contrary, when the %ARfD value surpasses 100, the potential danger appears impermissible. As %ARfD levels climb, the more severe the brief dietary vulnerability becomes.

In the risk assessment process, for substances whose toxicity manifests only after long-term repeated exposure, chronic dietary exposure assessment is considered a necessary component. Typically, the proportion between International Estimated Daily Intake (IEDI) and ADI, calculated through theoretical methods, is used to represent this. When calculating IEDI, the JMPR combines the supervised trials median residue (STMR) levels from pesticide residue monitoring tests with dietary clustering information of the population. To more accurately understand dietary exposure, following WHO recommendations [23], in the lower bound (LB) assessment (best-case scenario), pesticide residue values below the Limit of Quantification (LOQ) are considered as zero, while in the upper bound (UB) assessment (worst-case scenario), they are considered as the LOQ.

(4) $$IEDI=\frac{x\times c}{b_{w}}$$

(5) $$\%ADI=\frac{IEDI}{ADI}\times 100\%$$

Here, %ADI denotes the sustained dietary exposure threat; x reflects the general consumption of pineapple by the population; and c is the pesticide residue value. The consumption information derives from the GEMS/Food contaminants database [20]. The ADI figure originates from the Chinese National Standard GB 2763 [18]. If %ADI remains below 100, the potential long-term hazard becomes tolerable. However, when the %ADI value exceeds 100, the potential risk is deemed unacceptable. Thus, rising %ADI levels suggest enhanced sustained exposure threat.

Following the studies of Reffstrup [24] and Eylem Odabas [25], the HI is adopted to measure the overall vulnerability from pesticide exposure. The calculation formula is as follows:

(6) HI=∑%ADI

## 3. Results and Discussion

### 3.1. Method Performance and Quality Assurance

In order to assess the reliability of the established method, various standard solutions were spiked into pesticide-free pineapple samples and analyzed using the aforementioned procedure. According to the China method validation guidelines GB/T 27417 [26], the reliability of the method was validated by indicators such as recovery rate, detection limit (LOD), quantification limit (LOQ), and precision, and the liquid chromatography–mass spectrometry system was linearly validated. The related results are detailed in Table A1. Regression coefficients exhibited r^2^ values beyond 0.99, as evidenced by our analysis. The recovery rates for spiked standard pesticides ranged from 76.4% to 119.5%. The relative standard deviations (RSD) for all pesticides were below 15%. The LOD and LOQ ranged from 0.0002 mg/kg to 0.01 mg/kg and from 0.0006 mg/kg to 0.03 mg/kg, respectively. The lowest signal-to-noise ratio (S/N) for detection was 3, and the lowest spiking level provided satisfactory recovery (70–120%) with an RSD less than 20%. In order to ensure the accuracy of the results of each batch of samples, the standard solution of pesticide was added to the blank sample as the quality control sample. When the recovery rate of the quality control sample was 70–120%, the results of the batch of samples were considered accurate and reliable.

### 3.2. Pesticide Residue Levels in Pineapples

**Figure 2:**
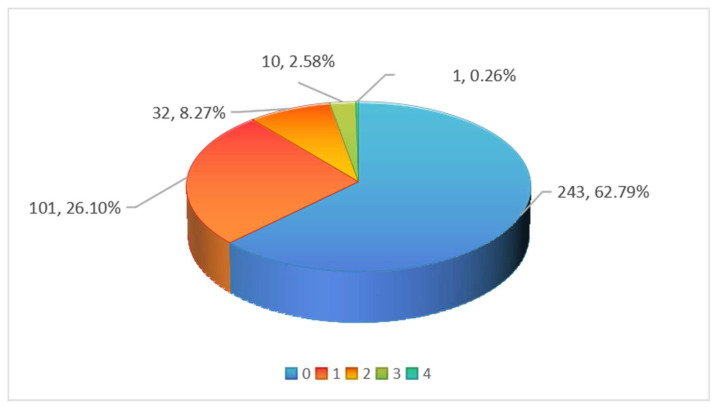
Multiple pesticide detections in pineapple samples.

As shown in Figure 2, among the 387 batches of pineapple samples, 144 batches (37.21%) had pesticide residues detected, with 11.11% of the samples containing residues of two or more pesticides. This indicates that multiple pesticides or pesticide formulations with various active ingredients are used during pineapple cultivation.

In Table 2, pesticide contamination concentrations within pineapples are described in greater detail, including 11 insecticides, 8 fungicides, and 1 plant growth regulator. The detection rates exhibit a three-tier distribution. Gibberellic acid, cypermethrin, and carbendazim are in the first tier, with higher detection rates exceeding 10%; the second tier includes pesticides like methyl thiophanate, cyhalothrin, and myclobutanil, with detection rates ranging from 1% to 5%; while the third tier includes pesticides such as tebuconazole and thiamethoxam, with detection rates below 1% in some samples. Based on the Chinese National Standard GB2763 [18], 5 out of the 387 batches exceeded the MRLs, including cyhalothrin, thiamethoxam, and carbendazim, resulting in a compliance rate of 98.71%.

Compared with other fruits, both the frequency and quantity of pesticide contamination found within pineapples are relatively low. Tao Pei et al. [27] analyzed the residues of 102 pesticides in apples and found that 34 types of pesticides were present, with the detection rates of carbendazim, tebuconazole, and acetamiprid exceeding 50%. Lixue Kuang et al. [28] investigated residues of 57 pesticides in lychee and longan, with pesticide contamination identified in 72% of examined lychee materials and 52% of examined longan materials. Contrary to Tarık Balkan et al.’s findings, no pesticide residues were detected in the pineapple samples [29], likely due to the small sample size.

### 3.3. Risk Ranking

**Figure 3:**
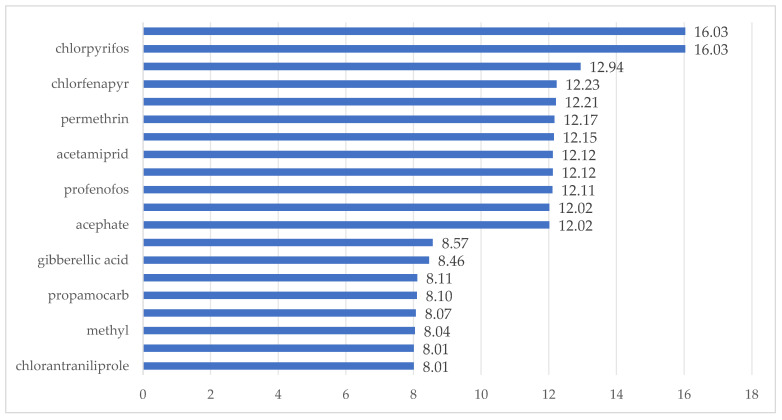
Risk ranking of pesticide residues.

Based on the average food consumption and fruit consumption per capita of Chinese residents [30] and pineapple production data [4], it is estimated that the dietary intake of pineapples accounts for less than 2.5% of the total diet. Accordingly, the dietary proportion score (C) for pineapples, as determined in Table 1, is 0. Adhering to principles of prudent pesticide application and common application practices, each pesticide is applied up to three times during the pineapple growth period. Typically, it takes 120–150 days for Bali pineapple varieties to go from flowering to fruiting; thus, the estimated pesticide application frequency is less than 2.5%, resulting in a pesticide use frequency score (D) of 0. In China, there is currently no relevant information available on high-exposure populations, so the high-exposure population score (E) is 3. Based on Formula (2), the scores for the 20 pesticides are shown in Figure 3. According to the risk classification rules outlined by Yao Qinghua et al. [31], among the 20 pesticides, chlorpyrifos and cyhalothrin are classified as high-risk pesticides, while the remaining 18 pesticides have risk scores below 15%, falling into the low-risk category.

**Figure 4:**
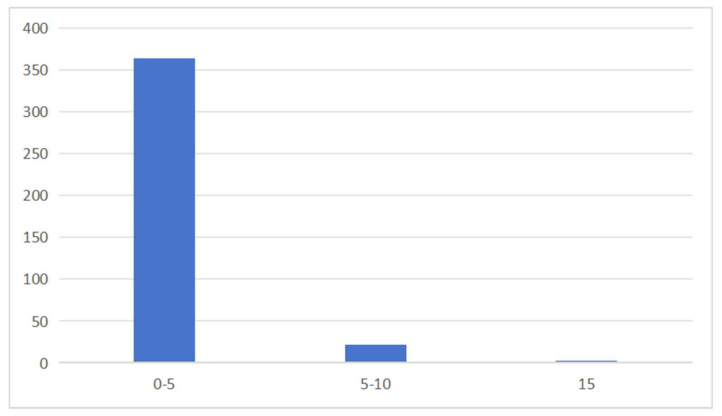
Risk level distribution of samples.

According to the classification criteria presented by Nie Jiyun et al. [16], an RI ≥ 15 is classified as high risk, 10 ≤ RI < 15 as medium risk, 5 ≤ RI < 10 as low risk, and RI < 5 as extremely low risk. Based on the calculation using Formula (6), among the 387 batches of samples, only 1 sample had an RI greater than 15, making it a high-risk sample, accounting for 0.26% of the total. There were 22 samples with an RI between 5 and 10, representing 5.68% of the total, while the remaining 364 batches had an RI below 5, which were classified as extremely low risk, making up 94.06% of the samples (Figure 4).

#### 3.3.1. Short-Term Intake and Acute Exposure Risk

MRLs are human-set thresholds and do not directly reflect toxicological hazards. Influenced by Good Agricultural Practices (GAP) and trade factors, MRL values tend to be conservative and stricter than toxicological risk thresholds. Therefore, pesticide contamination detection beyond MRL thresholds within crops fails to necessarily suggest health hazards. Instead, dietary exposure risk assessments should be conducted using toxicological benchmarks, such as ADI values and ARfD parameters.

**Figure 5:**
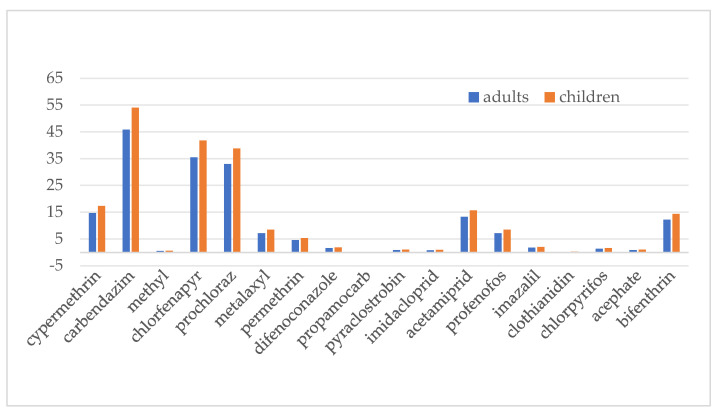
Acute dietary exposure risk assessment.

In line with JMPR protocols, risks from acute exposure to potential hazardous factors through short-term food consumption must be evaluated [22]. As the JMPR does not have ARfD data for chlorantraniliprole and gibberellic acid, this study compares short-term food intake hazards for the other 18 chemical compounds across various demographic segments. As can be seen from Figure 5, the acute dietary exposure risk for adults ranges from 0.19 to 45.83, while for children, the risk ranges from 0.22 to 53.98, with children’s acute dietary exposure risk being higher than that of adults. Among the 18 pesticides, carbendazim has the highest acute dietary exposure risk, followed by cyhalothrin and mefenoxam, with their %ARfD around 40%. The second-tier group includes six pesticides—cypermethrin, metalaxyl, permethrin, acetamiprid, profenofos, and bifenthrin—with %ARfD around 10. The remaining nine pesticides fall into the third tier, with %ARfD around 1. Overall, the acute dietary exposure risk from pesticide residues in pineapples remains within an acceptable range.

#### 3.3.2. Long-Term Intake and Chronic Dietary Exposure Risk

**Figure 6:**
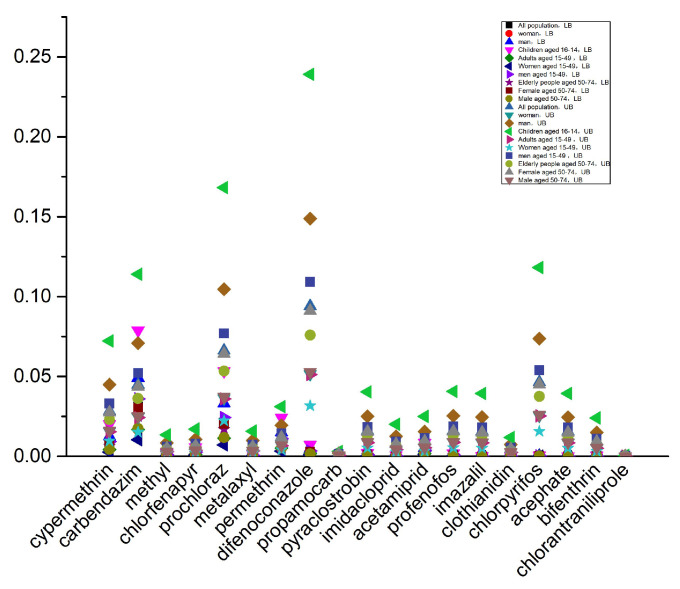
Chronic dietary exposure risk assessment under LB and UB conditions.

**Figure 7:**
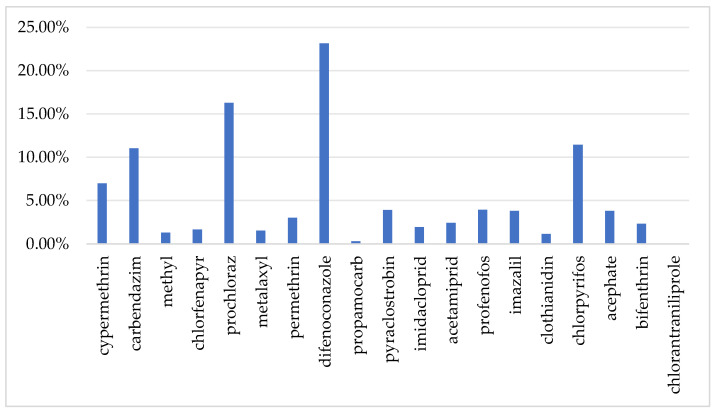
Contribution of detected pesticides to the HI.

Gibberellic acid is a plant endogenous growth regulator, which is typically considered safe and does not require the establishment of a residue limit standard [32], nor does it have an ADI. Findings from chronic food intake safety evaluation for the remaining 19 agrochemicals in different population subgroups across optimistic and pessimistic conditions appear in Figure 6. When using a mean-value distribution model, under the worst-case scenario, the %ADI for all pesticides is below 1. Furthermore, among the 10 population subgroups, the long-term dietary exposure risk for children is higher than that for other subgroups. This may be due to higher pineapple consumption levels and lighter body weight in children. In the worst-case scenario, the HI values for the 10 population subgroups range from 0.03% to 1.03%, with the highest contribution from difenoconazole at 23.13%, followed by carbendazim, prochloraz, and chlorpyrifos, each contributing over 10% (Figure 7). According to a report from the European Food Safety Authority (EFSA), chronic dietary risks are considered significant only when exposure levels approach or exceed 100% of the ADI [33]. In this context, the pesticide levels detected in pineapples in China are far below the risk threshold, posing no significant chronic health risk to consumers.

### 3.4. Uncertainty and Limitations

Although the results of this study show that the compliance rate for pesticide residues in pineapples is relatively high (based on China’s pesticide residue limits) and that both acute and chronic dietary exposure risks are within acceptable limits, there are still some uncertainties. This is primarily due to the fact that only 88 pesticides were monitored in this study, covering most of the pesticides used in production, but some pesticides were not included in the monitoring scope, leading to unknown risks for those pesticides. Additionally, due to the lack of dietary consumption data for pineapples in China, the study referenced pineapple consumption data from Australia for adults and from Japan for children. Furthermore, the risk calculation formulas for children and adults are not consistent, which may lead to deviations in the actual acute food intake hazards associated with pineapples within China compared to this study. Another limitation is that pineapples are typically consumed after peeling, but this study measured pesticide residues in the entire pineapple (with peel), which may result in an overestimation of the dietary risk of pineapples. Although replacing non-detected values with the LOQ or zero is a common practice in risk assessments, this approach introduces uncertainty in the risk calculation. Therefore, future research should develop more sensitive detection methods to accurately reflect the actual pesticide residue levels in the samples.

## 4. Conclusions

Based on continuous monitoring results over three years, 20 chemical compounds were found among pineapples in China. The predominantly found agrochemicals were cypermethrin, carbendazim, and gibberellic acid, which are used for insecticide, fungicide, and fruit enlargement purposes, respectively. When sorted by residue risk score, chlorpyrifos and cypermethrin were found to pose the highest risk and should be given special attention. Using the risk index to rank the pesticide residue risks in samples, 99.74% of the samples were found to fall into the medium, low, or very low-risk categories. Through chronic dietary risk assessment and short-term dietary risk evaluation, in the worst-case scenario, the ADI % per individual pesticide in the 10 population subgroups was below 1%, and the %ARfD was below 60%. The HI distribution ranged from 0.03% to 1.03%, with children’s chronic dietary risk being higher than that of adults. These findings indicate that the chronic dietary exposure risk from pineapples is acceptable and does not pose a significant health risk.

## Figures and Tables

**Table 1:** Score of 6 indices for risk ranking of pesticide residues in pineapple.

Index	Index Value	Score	Index Value	Score	Index Value	Score	Index Value	Score
Toxicity	Low	2	Moderate	3	High	4	Very high	5
Potency (mg·kg^−1^)	>1 × 10^−2^	0	1 × 10^−4^–1 × 10^−2^	1	1 × 10^−6^–1 × 10^−4^	2	<1× 10^−6^	3
Share of pineapple in diet (%)	<2.5	0	2.5–20	1	20–50	2	50–100	3
Frequency of dosing (%)	<2.5	0	2.5–20	1	20–50	2	50–100	3
High exposure group	No	0	Unlikely	1	Likely	2	Existing or no data to judge	3
Residue level (mg·kg^−1^)	Nd	1	<1 MRL	2	≥1 MRL	3	≥10 MRL	4

**Table 2:** Pesticide residue levels in pineapple samples.

Pesticide	Toxicity	Minimum Value/mg/kg	Maximum Value/mg/kg	Detection Rate/%	Average Value/mg/kg		MRL/mg/kg	Over-Standard Rate/%	ADI/mg/kg
					LB	UB			
cypermethrin	moderate toxicity	0.01	0.082	11.63	0.00341	0.01224	/	/	0.02
carbendazim	low toxicity	0.015	1.28	10.59	0.02005	0.02899	0.5	0.26	0.03
gibberellic acid	low toxicity	0.11	0.13	11.83	0.00530	0.01297	/	/	/
Methyl thiophanate	low toxicity	0.021	0.069	3.8	0.00165	0.01026	/	/	0.09
chlorfenapyr	moderate toxicity	0.012	0.099	2.84	0.00096	0.00290	0.05	0.78	0.02
prochloraz	low toxicity	0.011	0.92	2.58	0.00452	0.01426	7	0	0.01
metalaxyl	low toxicity	0.011	0.5	2.07	0.00095	0.01074	/	/	0.08
permethrin	moderate toxicity	0.088	0.95	2.07	0.01025	0.01319	2	0	0.05
difenoconazole	low toxicity	0.013	0.066	1.81	0.00063	0.02027	0.2	0	0.01
propamocarb	low toxicity	0.016	0.053	1.81	0.00056	0.01037	/	/	0.4
pyraclostrobin	low toxicity	0.012	0.082	1.79	0.00053	0.01026	1	0	0.03
imidacloprid	moderate toxicity	0.011	0.043	1.55	0.00036	0.01020	/	/	0.06
acetamiprid	moderate toxicity	0.021	1.3	1.55	0.00497	0.01481	2	0	0.07
profenofos	moderate toxicity	0.025	1	1.29	0.00046	0.01033	/	/	0.03
imazalil	moderate toxicity	0.012	0.012	0.26	0.00004	0.01001	/	/	0.03
clothianidin	low toxicity	0.017	0.017	0.26	0.00006	0.01002	0.01	0.26	0.1
chlorpyrifos	moderate toxicity	0.019	0.019	0.26	0.00005	0.01002	/	/	0.01
acephate	moderate toxicity	0.012	0.012	0.26	0.00003	0.01001	0.02	0	0.03
bifenthrin	moderate toxicity	0.017	0.017	0.26	0.00004	0.00204	/	/	0.01
chlorantraniliprole	low toxicity	0.1	0.1	0.26	0.00026	0.01023	/	/	2

## Data Availability

The raw data supporting the conclusions of this article will be made available by the authors on request.

## Abbreviations

The following abbreviations are used in this manuscript:

ADI	Acceptable Daily Intake
ARfD	Acute Reference Dose
MRLs	Maximum Residue Limits
LB	Lower Bound
LOQ	Limit of Quantification
UB	Upper Bound
IEDI	International Estimated Daily Intake
LOD	Limit of Detection
RSD	Relative Standard Deviations
RI	Risk Index
FPD	Flame Photometric Detector
ECD	Electron Capture Detector
GC	Gas Chromatography
GC-MS	Gas Chromatography–Mass Spectrometry
LC-MS/MS	Liquid Chromatography–Mass/Mass Spectrometry

## Appendix A

**Table A1:** The linear regression equation, correlation coefficient, LOD, LOQ, recovery, and RSD for the targeted compounds.

Group	Compound	Equation	R^2^	Monitoring Ion	LOD (mg/kg)	LOQ (mg/kg)	Recovery (%)	RSD (%)
14	acephate	/	/	/	0.003	0.01	112.7	1.2
	omethoate	/	/	/	0.003	0.01	91.4	12.1
	phorate	/	/	/	0.003	0.01	78.0	9.3
	malathion	/	/	/	0.003	0.01	80.7	12.4
	chlorpyrifos	/	/	/	0.003	0.01	95.0	14.9
	isofenphos-methyl	/	/	/	0.003	0.01	76.5	7.5
	phosfolan	/	/	/	0.003	0.01	106.6	12.9
	phosmet	/	/	/	0.003	0.01	88.7	8.5
	methamidophos	/	/	/	0.003	0.01	97.4	3.7
	dimethoate	/	/	/	0.003	0.01	113.5	10.3
	methyl-parathion	/	/	/	0.003	0.01	77.6	14.9
	isocarbophos	/	/	/	0.003	0.01	117.0	10.3
	triazophos	/	/	/	0.003	0.01	82.5	1.5
	dichlorvos	/	/	/	0.01	0.03	115.7	9.3
	ethoprophos	/	/	/	0.003	0.01	95.5	2.8
	diazinon	/	/	/	0.003	0.01	114.8	6.5
	fenitrothion	/	/	/	0.003	0.01	92.0	12.9
	phorate sulfoxide	/	/	/	0.003	0.01	106.2	4.5
	phorate sulfone	/	/	/	0.003	0.01	109.1	12.0
	parathion	/	/	/	0.003	0.01	100.1	2.9
	profenofos	/	/	/	0.003	0.01	114.7	10.5
	phosalone	/	/	/	0.003	0.01	87.7	0.4
	trichlorfon	/	/	/	0.003	0.01	118.1	5.5
2	chlorothalonil	/	/	/	0.0003	0.001	106.2	11.4
	triadimefon	/	/	/	0.001	0.003	92.0	12.9
	procymidone	/	/	/	0.002	0.006	96.8	3.3
	bifenthrin	/	/	/	0.0006	0.002	87.1	11.5
	fenpropathrin	/	/	/	0.002	0.006	94.1	12.4
	chlorfenapyr	/	/	/	0.0005	0.002	85.3	11.1
	permethrin	/	/	/	0.001	0.003	110.9	8.7
	cypermethrin	/	/	/	0.003	0.01	102.4	10.8
	fenvalerate	/	/	/	0.002	0.006	89.9	2.9
	deltamethrin	/	/	/	0.001	0.003	110.6	11.4
	pcnb	/	/	/	0.0002	0.0006	88.9	11.5
	vinclozolin	/	/	/	0.0004	0.0015	102.1	14.2
	dicofol	/	/	/	0.0008	0.0025	89.4	11.3
	iprodione	/	/	/	0.0003	0.001	119.0	2.0
	cyfluthrin	/	/	/	0.00065	0.002	103.3	9.6
	flucythrinate	/	/	/	0.0003	0.001	117.3	3.9
	fluvalinate	/	/	/	0.0003	0.001	84.3	2.1
	six six six	/	/	/	0.0002	0.0008	87.4	12.6
3	carbofuran	y = 14,571.7*x − 11,791.3	0.999	222.1 > 123, 22.1 > 165.1	0.003	0.01	112.0	11.73
	3-hydroxy carbofuran	y = 5271.98*x − 4049.79	0.999	238.1 > 181.1, 238.1 > 163.1	0.003	0.01	116.0	2.1
	imidaclopnd	y = 4151.25*x − 8651.38	0.992	256.1 > 209.1, 256.1 > 175.1	0.003	0.01	98.0	2.2
	carbendazim	y = 43,253.7*x − 10,335.5	0.993	192.1 > 160.1, 192.1 > 132.1	0.003	0.01	89.5	0.9
	propamocarb	y = 765,937.9*x − 167,535	0.996	189.2 > 102.1, 189.2 > 74	0.003	0.01	78.7	5.1
	forchlorfenuron	y = 73,109.55*x − 6040.25	0.996	248.1 > 129, 248.1 > 93	0.003	0.01	102.3	13.4
	chlorantraniliprole	y = 673.063*x − 628.472	0.998	484 > 452.9, 484 > 285.9	0.003	0.01	88.6	12.5
	tebufenozide	y = 8529.54*x + 840.371	0.994	353.2 > 133.1, 353.2 > 297.2	0.003	0.01	80.4	13.3
	aldicarb	y = 553.017*x − 962.568	0.996	208.1 > 116.1, 208 > 89	0.003	0.01	115.1	5.2
	aldicarb sμlfone	y = 6329.04*x − 230.722	0.997	240.1 > 148, 240.1 > 166.1	0.003	0.01	107.6	5.9
	aldicarb sμlfoxide	y = 16,832.9*x − 9636.43	0.999	207.1 > 132, 207.1 > 89	0.003	0.01	81.0	0.9
	phoxim	y = 4601.63*x − 8151.81	0.995	299.1 > 129, 299.1 > 153	0.003	0.01	106.0	2.5
	methomyl	y = 5661.3*x − 1208.61	0.999	163.1 > 88, 163.1 > 106	0.003	0.01	103.1	4.3
	carbaryl	y = 4178.66*x − 1739.25	0.999	202.1 > 127.1, 202.1 > 145.1	0.003	0.01	112.6	9.7
	abamectin	y = 28.601*x − 34.0807	0.993	895.5 > 751.4, 895.5 > 449.2	0.003	0.01	99.4	7.8
	chlorbenzuron	y = 1605.21*x + 1205.39	0.994	309 > 156, 309 > 139	0.003	0.01	113.8	13.2
	diflubenzuron	y = 2483.15*x − 2866.46	0.997	311 > 158, 311 > 141	0.003	0.01	111.6	3.7
	acetamipnd	y = 11,271.2*x − 4561.67	0.999	223.1 > 126, 223.1 > 99	0.003	0.01	112.0	8.7
	thiamethoxam	y = 5136.49*x − 4601.22	0.999	292 > 211.1, 292 > 181.1	0.003	0.01	99.1	5.3
	paclobutrazol	y = 2750.28*x − 3851.37	0.990	294.1 > 70, 294.1 > 125	0.003	0.01	107.7	11.1
	prochloraz	y = 6405.53*x − 4868.47	0.999	376.2 > 308, 376.2 > 266	0.003	0.01	112.3	6.6
	dimethomorph	y = 4352.63*x − 7750.02	0.997	388.1 > 165.1, 388.1 > 301.1	0.003	0.01	100.3	14.7
	azoxystrobin	y = 322.87*x + 221.747	0.997	404.1 > 372.1, 404.1 > 344.1	0.003	0.01	77.4	3.9
	chlorfluazuron	y = 887.344*x − 1040.55	0.997	542 > 384.9, 540 > 382.9	0.003	0.01	84.6	8.9
	cyromazine	y = 1063.87*x − 3128.38	0.991	167 > 125, 167 > 108	0.003	0.01	119.1	2.1
	pyraclostrobin	y = 10,546*x − 7790.48	0.998	388.1 > 194.1, 388.1 > 163.1	0.003	0.01	100.2	13.5
	emamectin benzoate	y = 17,047.7*x − 17,828.6	0.999	886.5 > 158.1, 886.5 > 82.1	0.003	0.01	119.5	11.3
	demeton	y = 5261.8*x − 12,391.8	0.993	259.1 > 89, 259.1 > 61	0.003	0.01	85.9	2.1
	isazofos	y = 2775.26*x − 2339.38	0.998	316 > 122, 316 > 164	0.003	0.01	105.0	13.2
	propiconazole	y = 2157.04*x − 800.439	0.999	342.1 > 159, 344.1 > 161	0.003	0.01	108.2	6.4
	carbosμLfan	y = 5534.3*x − 457.309	0.995	381.221 > 118, 381.221 > 76	0.003	0.01	76.4	14.0
	spinosad A	y = 13,087.6*x − 27,823	0.995	732.5 > 142, 732.5 > 98.1	0.003	0.01	111.7	10.9
	spinosad D	y = 15,626.7*x − 9693.53	0.999	746.5 > 142.1, 746.5 > 98	0.003	0.01	77.0	4.7
	tebuconazole	y = 6027.11*x − 1272.18	0.999	308.2 > 70, 308.2 > 125	0.003	0.01	92.2	3.8
	spinetoram (J)	y = 14,909.2*x − 24459.4	0.993	748.5 > 142.1, 748.5 > 203.1	0.003	0.01	114.8	10.0
	spinetoram (L)	y = 13,980.3*x − 3905.21	0.998	760.5 > 142.1, 760.5 > 98.1	0.003	0.01	78.4	12.6
	clothianidin	y = 771.584*x + 286.078	0.995	250 > 169.1, 250 > 132	0.003	0.01	94.2	12.6
	thidiazuron	y = 915.937*x − 2480.16	0.992	221 > 102, 221 > 128	0.003	0.01	90.0	5.2
	imazalil	y = 3410.58*x − 4049.38	0.998	297.1 > 159, 297.1 > 201	0.003	0.01	110.3	3.0
	thiabendazole	y = 20,004.7*x − 14,397.9	0.999	202 > 175, 202 > 131.1	0.003	0.01	92.8	9.1
	thiophanate-methyl	y = 6281.99*x + 18,736.3	0.994	343.1 > 151, 343.1 > 311	0.003	0.01	84.0	14.5
	myclobutanil	y = 3339.81*x + 1730.75	0.996	289.1 > 70, 289.1 > 125	0.003	0.01	108.4	12.2
	gibberellic acid	y = 7.91779*x − 13.2817	0.992	345 > 239, 345 > 143	0.003	0.01	83.8	12.6
4	fipronil	/	/	367, 369, 351	0.003	0.01	84.7	5.9
	fluoroacetonitrile	/	/	333, 388, 390	0.003	0.01	87.6	5.1
	fipronil sulfide	/	/	351, 353, 420	0.003	0.01	104.1	5.9
	fluconazole sulfone	/	/	383, 385, 452	0.003	0.01	107.1	13.7
	pyridaben	/	/	147, 117, 364	0.001	0.003	78.7	1.8
	difenoconazole	/	/	323, 267, 325	0.006	0.02	113.8	0.8
	pyrimethanil	/	/	198, 199, 200	0.003	0.01	76.7	2.9
	chlorfenapyr	/	/	247, 328, 408	0.01	0.03	94.5	1.2
	pendimethalin	/	/	252, 162, 220	0.003	0.01	76.9	13.3
	metalaxyl	/	/	206, 249, 234	0.003	0.01	84.3	14.6
	etofenprox	/	/	163, 183, 376	0.001	0.003	93.4	13.9
	fenthion	/	/	278, 169, 245	0.003	0.01	114.8	12.9
	fenthion sulfone	/	/	310, 136, 231	0.01	0.03	85.6	7.3
	fenthion-sulfoxide	/	/	279, 294, 278	0.01	0.03	82.0	9.4

Note: Except for the third group of pesticides, all other groups of pesticides were calibrated using the single-point method.
